# Associations of Inflammatory Markers and Coronary Heart Disease in Different Gender Groups in Cohort NHANES 2003–2018

**DOI:** 10.1155/crp/5555602

**Published:** 2026-02-03

**Authors:** Anmin Ren, Qianjun Liu, Qian Gan, Liming Lu, Xin Kai Qu

**Affiliations:** ^1^ Department of Cardiology, Huadong Hospital, Fudan University, Shanghai, China, fudan.edu.cn; ^2^ Department of Gerontology, Huadong Hospital, Fudan University, Shanghai, China, fudan.edu.cn; ^3^ Shanghai Key Laboratory of Clinical Geriatric Medicine, Huadong Hospital, Fudan University, Shanghai, China, fudan.edu.cn; ^4^ Shanghai Jiao Tong University School of Medicine, Shanghai, China, shsmu.edu.cn

**Keywords:** coronary heart disease, inflammatory markers, NHANES

## Abstract

**Background:**

Although previous studies have revealed the correlation between inflammatory markers and coronary heart disease (CHD), this study aims to explore the relationship between inflammatory markers and CHD in the male and female population, respectively.

**Methods:**

This study includes participants from the National Health and Nutrition Examination Survey (NHANES) from 2003 to 2018. Inflammatory markers included the following: systemic immune‐inflammation index (SII), lymphocyte‐to‐monocyte ratio (LMR), neutrophil‐to‐lymphocyte ratio (NLR), and platelet‐to‐lymphocyte ratio (PLR). Multivariate logistic regression was performed to investigate the correlation between these inflammatory markers and CHD. The trend test was employed to examine potential linear trend associations, and the restricted cubic splines (RCSs) were utilized to depict nonlinear relationships.

**Results:**

The NHANES database including 40,177 participants was stratified into two cohorts: the CHD group (*n* = 1667) and the non‐CHD group (*n* = 38,510). With further gender stratification, we found that LMR, PLR, and SII all exhibited negatively significant correlation with CHD in the male group, while LMR and NLR were meaningful factors in the female group. We also detected that LMR, PLR, and SII all have nonlinear relationship with CHD in the male group (*p* for nonlinear < 0.05), while PLR had nonlinear relationship with CHD in the female group (*p* for nonlinear < 0.05).

**Conclusions:**

Our study revealed that LMR, PLR, and SII are significantly negative correlative markers of CHD in males, while LMR and NLR are more accurate predictors of CHD in females.

## 1. Introduction

Coronary heart disease (CHD) stands as the foremost contributor to global mortality and morbidity, influenced by a complex interplay of genetic, lifestyle determinants [1]. In 2020, CHD (41.2%) is projected to be the leading cause of cardiovascular disease–related mortality in the United States, followed by stroke (17.3%), a combination of other minor cardiovascular diseases (16.8%), high blood pressure (12.9%), heart failure (9.2%), and arterial disease (2.6%) [2]. Meanwhile, the prevalence rates varied among different gender groups. In the United States, from 1994 to 2020, there was a reduction in CHD mortality for both genders, and the decline was less pronounced among young women compared to that of young men [3]. Therefore, identification of factors contributing to the gender disparity in CHD will be beneficial to the development of CHD management strategy.

Injury and inflammatory processes play an important role in the development of atherosclerotic plaque which is a primary contributor to CHD [4]. The migration of leukocytes from the bloodstream to tissues is a characteristic feature of inflammation and the defense of the host against microbial pathogens [5]. Neutrophils, monocytes, lymphocytes, and platelets each fulfill distinct functions within the inflammatory cascade [6]. Therefore, inflammation markers are often associated with cardiovascular disease. Previous studies have shown that multiple inflammatory markers, including neutrophil‐to‐lymphocyte ratio (NLR), systemic immune‐inflammation index (SII), lymphocyte‐to‐monocyte ratio (LMR), and platelet‐to‐lymphocyte ratio (PLR), are highly correlated with the occurrence of CHD [7–10]. However, few studies examined the combinatorial prognostic efficacy of LMR, NLR, PLR, and SII in a cohort of CHD participants. Previous research has identified a curvilinear association between SII and CHD in both male and female populations [8]. Ting et al.​ reported a nonlinear relationship between LMR and the incidence of heart failure in males [11]. Taken together, it is essential to explore the predictive effect of these inflammatory indicators in CHD populations of different genders.

In this study, a cross‐sectional US population utilizing sample from the National Health and Nutrition Examination Survey (NHANES) was conducted to investigate the relationship between above inflammatory markers and CHD prevalence among different sex groups. This study uses multiple statistical strategies to show that LMR, PLR, and SII are significant correlative markers of CHD in males, while LMR and NLR are more accurate predictors of CHD in females. In summary, our work has identified different inflammatory markers that correlate with the disease risk in males and females, respectively.

## 2. Methods

### 2.1. Study Population

NHANES is a cross‐sectional study conducted by the National Center for Health Statistics (NCHS) to comprehensively investigate the health and nutrition status of a representative sample of the US population [12]. The NHANES database is based on a 2‐year survey cycle. These study data were from eight 2‐year survey cycles, from 2003 to 2018. All participants in the NHANES survey completed the questionnaire and signed the provided informed consent form. Additional detailed information about NHANES can be found on the website: https://www.cdc.gov/nchs/nhanes/.

**Figure 1:**
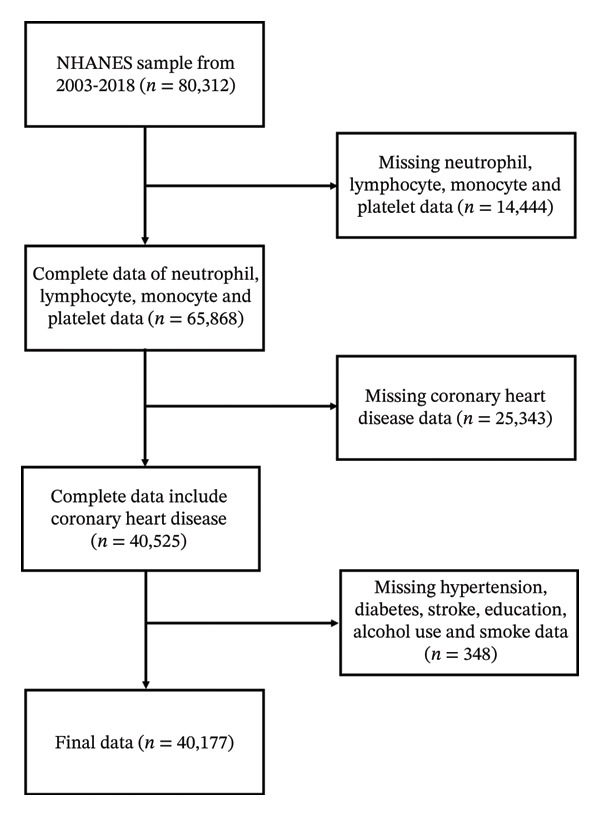
Flow chart of study participants. NHANES, National Health and Nutrition Examination Survey.

The study recruited 80,312 subjects over eight cycles, after excluding 14,444 subjects with missing neutrophil, lymphocyte, monocyte, and platelet data; 25,343 subjects with missing CHD data; and 348 subjects with missing covariates: hypertension, diabetes, stroke, education, alcohol use, and smoke data; the study ultimately encompassed 40,177 eligible participants (Figure 1).

### 2.2. Assessment of CHD

CHD verification relied on the questionnaire (MCQ_I), which included the query “Ever told you had coronary heart disease?” Participants who responded “YES” were considered to be diagnosed with CHD [13].

### 2.3. Detection of Inflammation Markers

The methodologies employed for determining complete blood count (CBC) parameters are founded on the Beckman–Coulter approach to enumeration and sizing. The CBC was performed using an automated hematology analyzer. The inflammation markers were derived from the CBC test results, which based on these formulas: SII = platelet count × neutrophil count/lymphocyte count, NLR = neutrophils = lymphocytes, PLR = platelets/lymphocytes, and LMR = lymphocytes/monocytes [11].

### 2.4. Covariate Definition

Our study selected several covariates that could potentially impact the relationship between inflammation markers and CHD. The demographic and health questionnaires of the NHANES survey include the following: age (years), gender (male/female), education, race (Mexican American/non‐Hispanic White/non‐Hispanic Black/other race), body mass index (BMI, kg/m^2^), smoked at least 100 cigarettes, alcohol use, hypertension, CHD, congestive heart failure, and stroke. Subjects fulfilling one or more of the following criteria are categorized as having diabetes: (1) fasting blood glucose ≥ 7.0 mmol/L or a 2‐h oral glucose tolerance test result ≥ 11.1 mmol/L; (2) random blood glucose ≥ 11.1 mmol/L; (3) hemoglobin A1c (HbA1c) ≥ 6.5%; (4) currently using antidiabetic drugs or insulin; and (5) diagnosis of diabetes by a physician [14]. Educational level was categorized as college level or higher (> high school), high school or its equivalent, and below high school (< high school) [15]. Smoker was defined as individuals who had a history of smoking at least 100 cigarettes in their lifetime [16]. Active alcohol user was defined as having at least 12 drinks per year, while nonactive alcohol user was defined as having fewer than 12 drinks per year [17]. Some additional covariates, such as heart rate (HR), systolic blood pressure (SBP, mmHg), diastolic blood pressure (DBP, mmHg), glycated hemoglobin (HbA1c, %), blood cell counts (1000 cells/ul), urea nitrogen (UA, mg/dL), and serum creatinine (Scr, μmol/L), were all acquired from laboratory test results of the NHANES.

### 2.5. Statistical Analyses

The statistical analysis in this study employed weighted NHANES data and considered a complex multiperiod design. The baseline characteristic table for this study was stratified into two groups according to the presence or absence of CAD. The sampling weight was calculated as follows: WTINT2YR from demographic data/8. Continuous variables were expressed as mean ± SD, while normally distributed variables were analyzed using independent‐sample *t*‐tests, and non‐normally distributed variables were described using median with interquartile range and tested by Mann–Whitney U‐test. Categorical variables were represented by number and percentages, and their distribution was assessed using the chi‐square test. Univariate and multivariate logistic regression analyses were employed to identify significant covariates associated with inflammatory markers impacting CHD. This study utilized multivariate logistic regression models, including three different equations, to explore the association between inflammation markers and CHD: Model 1 (adjusted for no variables), Model 2 (adjusted for age and race), and Model 3 (adjusted for all covariates). The trend test (*p* for trend) is employed to examine the linear association between the variable and the CHD. Sex‐stratified logistic regression analysis was performed to determine whether these variables remained meaningful factors in the occurrence of CHD in different gender subgroups. A restricted cubic spline (RCS) was employed to investigate the nonlinear association with 4 knots in the male or female group. The RCS was adjusted for multiple covariates, including: age, race, HR, HbA1c, and the history of smoke, stroke, hypertension, diabetes, and congestive heart failure. The area under the curve (AUC) for SII, LMR, NLR, and PLR was determined using the receiver operating characteristic (ROC) curve, which assessed the predictive power of CHD. Statistical analyses were conducted using R (Version 4.3.0), along with Zstats v1.0 (https://www.zstats.net). A significance level of *p* < 0.05 was employed for all tests.

## 3. Results

### 3.1. Baseline Characteristics of Participants

This cross‐sectional study includes participants from the NHANES from 2003 to 2018. Table 1 includes the cohort of 40,177 participants, among which 1667 individuals were diagnosed with CHD, while the remaining 38,510 participants were identified as non‐CHD. The median age of all participants was 46 years, with 19,389 male and 20,788 female participants accounting for 47.9% and 52.1% of the study population. Significant differences in age, gender, race, education level, smoke, HR, HbA1c, UA, Scr, BMI, the history of diabetes, hypertension (SBP and DBP), congestive heart failure, and stroke were observed between patients with CHD and those without CHD (all *p* < 0.05). Table 1 presents the CHD population that exhibited significantly elevated white blood cell (*p* < 0.001), neutrophils (*p* < 0.001), lymphocyte (*p* < 0.001), and monocyte (*p* < 0.001) counts compared to the non‐CHD population while showing a significantly lower platelet value (*p* < 0.001). The PLR (*p* = 0.002) and NLR (*p* < 0.001) levels were significantly higher in CHD patients compared to non‐CHD patients, while the LMR (*p* < 0.001) was notably lower in CHD patients than in non‐CHD patients. No significant difference was found in SII levels between the non‐CHD and CHD groups (*p* = 0.274).

**TABLE 1:** Baseline characteristics of the study population between coronary heart disease (CHD) and non–coronary heart disease (non‐CHD).

	**Over all**	**CHD**	**Non-CHD**	***p* value**
	***n* = 40,177**	***n* = 1667**	***n* = 38,510**	

Age (y)	46.00 (33.00, 60.00)	68.00 (60.00, 76.00)	46.00 (33.00, 59.00)	< 0.001
Gender (%)				< 0.001
Male	19,389 (47.9)	1134 (66.0)	18,255 (47.3)	
Female	20,788 (52.1)	533 (34.0)	20,255 (52.7)	
Race (%)				< 0.001
Mexican American	6511 (8.4)	166 (3.6)	6345 (8.6)	
Non‐Hispanic White	17,492 (68.0)	1073 (81.3)	16,419 (67.5)	
Non‐Hispanic Black	8373 (10.9)	208 (5.9)	8165 (11.1)	
Other race	7801 (12.7)	220 (9.1)	7581 (12.8)	
Education (%)				< 0.001
< high school	10,156 (16.1)	530 (22.1)	9626 (15.9)	
high school	9295 (23.6)	384 (25.5)	8911 (23.5)	
> high school	20,726 (60.3)	753 (52.3)	19,973 (60.6)	
Smoked at least 100 cigarettes (%)				< 0.001
Yes	18,102 (45.4)	1055 (64.4)	17,047 (44.7)	
No	22,075 (54.6)	612 (35.6)	21,463 (55.3)	
Alcohol use (%)				0.073
Active alcohol user	30,325 (80.0)	1246 (77.7)	29,079 (80.1)	
Nonactive alcohol user	9852 (20.0)	421 (22.3)	9431 (19.9)	
BMI, kg/m^2^ (%)				< 0.001
< 25	11,528 (30.2)	361 (20.6)	11,167 (30.5)	
25–< 30	13,757 (33.1)	614 (34.2)	13,143 (33.1)	
> 30	14,892 (36.7)	692 (45.2)	14,200 (36.4)	
SBP (mmHg)	122.00 (112.00, 132.00)	128.00 (116.00, 142.00)	120.00 (112.00, 132.00)	< 0.001
DBP (mmHg)	70.00 (64.00, 78.00)	68.00 (58.00, 76.00)	72.00 (64.00, 78.00)	< 0.001
HR	72.00 (64.00, 80.00)	68.00 (60.00, 74.00)	72.00 (64.00, 80.00)	< 0.001
HbA1c (%)	5.40 (5.20, 5.70)	5.80 (5.50, 6.40)	5.40 (5.20, 5.70)	< 0.001
White blood cell, 1000 cells/μl	7.00 (5.80, 8.40)	7.20 (6.10, 8.60)	7.00 (5.79, 8.40)	< 0.001
Lymphocyte, 1000 cells/μl	2.00 (1.60, 2.50)	1.80 (1.40, 2.40)	2.00 (1.70, 2.50)	< 0.001
Neutrophils, 1000 cells/μl	4.10 (3.20, 5.20)	4.40 (3.40, 5.50)	4.10 (3.20, 5.20)	< 0.001
Monocyte, 1000 cells/μl	0.50 (0.40, 0.70)	0.60 (0.50, 0.70)	0.50 (0.40, 0.70)	< 0.001
Platelet, 1000 cells/μl	243.00 (207.00, 287.00)	211.00 (176.00, 257.00)	244.00 (208.00, 288.00)	< 0.001
UA (mg/dL)	5.30 (4.40, 6.30)	5.80 (5.00, 6.90)	5.30 (4.40, 6.30)	< 0.001
Scr (μmol/L)	76.02 (63.65, 88.40)	88.40 (74.26, 106.08)	75.14 (63.65, 88.40)	< 0.001
Diabetes (%)				< 0.001
Yes	5065 (9.3)	584 (33.2)	4481 (8.5)	
No	35,112 (90.7)	1083 (66.8)	34,029 (91.5)	
Hypertension (%)				< 0.001
Yes	14,266 (31.4)	1257 (73.2)	13,009 (29.9)	
No	25,911 (68.6)	410 (26.8)	25,501 (70.1)	
Congestive heart failure (%)				< 0.001
Yes	1312 (2.3)	528 (26.9)	784 (1.5)	
No	38,865 (97.7)	1139 (73.1)	37,726 (98.5)	
Stroke (%)				< 0.001
Yes	1546 (2.8)	282 (15.2)	1264 (2.4)	
No	38,631 (97.2)	1385 (84.8)	37,246 (97.6)	
LMR	3.80 (3.00, 4.80)	3.00 (2.40, 4.00)	3.83 (3.00, 4.83)	< 0.001
PLR	119.57 (95.22, 150.50)	114.29 (86.80, 150.00)	120.00 (95.45, 150.53)	0.002
NLR	2.00 (1.51, 2.62)	2.33 (1.73, 3.22)	1.97 (1.50, 2.61)	< 0.001
SII	482.00 (348.80, 675.54)	498.26 (342.24, 700.93)	481.50 (349.19, 674.67)	0.274

*Note:* Data are presented as number (%) or median (interquartile range). Non‐CHD: non–coronary heart disease; bA1c: glycosylated hemoglobin; HUA: urea nitrogen; Scr: serum creatinine; SII: systemic immune‐inflammation index.

Abbreviations: BMI, body mass index; CHD, coronary heart disease; DBP, diastolic blood pressure; HR, heart rate; LMR, lymphocyte‐to‐monocyte ratio; NLR, neutrophil‐to‐lymphocyte ratio; PLR, platelet‐to‐lymphocyte ratio; SBP, systolic blood pressure.

### 3.2. Association Between Inflammation Markers and CHD

Next, we examined the potential covariates that may affect CHD by univariate and multivariate logistic regression analysis [11]. The final analysis indicated that covariates, such as age, race, HR, HbA1c, and the history of smoke, stroke, hypertension, diabetes, and congestive heart failure, were significant contributing factors to the development of CHD (Table 2). A multimodel logistic regression analysis was employed to further investigate the correlation between inflammatory factors and CHD (Table 3). In Model 1 (unadjusted model), LMR, NLR, and SII were all significantly associated with CHD. In Model 2, which was adjusted for age, race, and gender, these four inflammatory indicators exhibited significant correlations with CHD, and the trend test revealed that all other indicators were statistically significant (all *p* for trend < 0.001), with the exception of SII. In Model 3 (adjusted for age, race, gender, and more covariates), during the Q3 stage, LMR showed a significant negative correlation with CHD (OR: 0.83, 95% CI: 0.71–0.97, *p* = 0.023). PLR was negatively correlated with CHD in all the stratified analyses: Q2, Q3, and Q4, and the *p* for trend was likewise significant (*p* for trend < 0.001). Meanwhile, significant positive associations with CHD were observed for SII and NLR, and the *p* for trend of SII was of significant effect (*p* for trend < 0.05).

**Table 2:** Results of univariable and multivariable logistic regression analyses between covariates and CHD.

Characteristics	Univariable analysis			Multivariable analysis		
	OR	95% CI	*p* value	OR	95% CI	*p* value
Age	1.08	1.08–1.08	< 0.001	1.05	1.05–1.06	< 0.001
Race						
Non‐Hispanic White	2.49	2.11–2.94	< 0.001	1.56	1.29–1.90	< 0.001
Non‐Hispanic Black	0.97	0.79–1.19	0.780	0.61	0.48–0.76	< 0.001
Other race	1.11	0.90–1.36	0.327	1.11	0.88–1.39	0.384
Gender	2.36	2.13–2.62	< 0.001	2.21	1.95–2.51	< 0.001
Education						
high school	0.78	0.68–0.90	< 0.001	0.91	0.78–1.06	0.227
> high school	0.68	0.61–0.77	< 0.001	1.01	0.88–1.16	0.875
BMI (kg/m^2^)						
25–< 30	1.45	1.27–1.65	< 0.001	0.98	0.84–1.13	0.768
> 30	1.51	1.32–1.72	< 0.001	1.06	0.91–1.24	0.427
HR	0.97	0.97–0.97	< 0.001	0.98	0.98–0.99	< 0.001
Alcohol use	0.96	0.86–1.08	0.483			
Smoke	5.99	1.96–2.40	< 0.001	1.43	1.27–1.61	< 0.001
HbA1c (%)	1.33	1.29–1.37	< 0.001	1.14	1.08–1.20	< 0.001
UA	1.33	1.29–1.38	< 0.001	1.00	0.96–1.04	0.929
Scr	1.01	1.00–1.01	< 0.001	1.00	1.00–1.00	0.285
Stroke	5.99	5.21–6.89	< 0.001	1.67	1.41–1.97	< 0.001
Hypertension	6.00	5.36–6.72	< 0.001	2.31	2.03–2.63	< 0.001
Diabetes	4.09	3.68–4.55	< 0.001	1.44	1.24–1.67	< 0.001
Congestive heart failure	22.29	19.66–25.26	< 0.001	8.10	7.01–9.36	< 0.001

*Note:* HbA1c: glycosylated hemoglobin; Scr: serum creatinine.

Abbreviations: BMI, body mass index; CHD, coronary heart disease; CI, confidence intervals; HR, heart rate; OR, odds ratio; UA, urea nitrogen.

**Table 3:** Univariate and multivariate analyses between LMR/PLR/NLR/SII and CHD in different models.

		**Model 1**		**Model 2**		**Model 3**	
		**OR (95% CI)**	***p* value**	**OR (95% CI)**	***p* value**	**OR (95% CI)**	***p* value**
LMR	Continuous	0.70 (0.67–0.73)	< 0.001	0.95 (0.92–0.99)	0.011	0.98 (0.95–1.02)	0.328
	Q2 (3.0–< 3.8)	0.49 (0.43–0.55)	< 0.001	0.82 (0.72–0.94)	0.004	0.92 (0.80–1.06)	0.242
	Q3 (3.8–< 4.8)	0.31 (0.27–0.36)	< 0.001	0.70 (0.60–0.81)	< 0.001	0.83 (0.71–0.97)	0.023
	Q4 (≥ 4.8)	0.25 (0.21–0.29)	< 0.001	0.75 (0.64–0.88)	< 0.001	0.86 (0.73–1.03)	0.095
	*p* for trend		< 0.001		0.007		0.183

PLR	Continuous	1.00 (1.00–1.00)	0.088	1.00 (0.99–1.00)	< 0.001	1.00 (1.00–1.00)	0.079
	Q2 (95.2–< 119.6)	0.74 (0.64–0.84)	< 0.001	0.76 (0.66–0.88)	< 0.001	0.82 (0.70–0.95)	0.010
	Q3 (119.6–< 150.5)	0.72 (0.63–0.83)	< 0.001	0.71 (0.61–0.82)	< 0.001	0.78 (0.67–0.91)	0.002
	Q4 (≥ 150.5)	0.89 (0.78–1.01)	0.066	0.68 (0.60–0.79)	< 0.001	0.75 (0.65–0.87)	< 0.001
	*p* for trend		0.001		< 0.001		< 0.001

NLR	Continuous	1.24 (1.20–1.27)	< 0.001	1.27 (1.19–1.36)	< 0.001	1.19 (1.10–1.27)	< 0.001
	Q2 (1.51–< 2.0)	1.20 (1.02–1.41)	0.030	1.05 (0.89–1.25)	0.552	1.02 (0.85–1.21)	0.857
	Q3 (2.00–< 2.6)	1.59 (1.37–1.85)	< 0.001	1.15 (0.98–1.35)	0.080	1.05 (0.88–1.24)	0.587
	Q4 (≥ 2.6)	2.54 (2.21–2.93)	< 0.001	1.33 (1.15–1.55)	< 0.001	1.07 (0.91–1.25)	0.432
	*p* for trend		< 0.001		0.003		0.563

SII	Continuous	1.00 (1.00–1.00)	< 0.001	1.00 (1.00–1.00)	0.022	1.00 (1.00–1.00)	0.002
	Q2 (348.1–< 482.0)	0.86 (0.75–0.99)	0.042	0.85 (0.73–0.98)	0.028	0.83 (0.71–0.97)	0.021
	Q3 (482.0–< 675.6)	1.03 (0.90–1.19)	0.625	0.96 (0.83–1.11)	0.575	0.93 (0.80–1.08)	0.353
	Q4 (≥ 675.6)	1.18 (1.03–1.35)	0.014	0.94 (0.82–1.09)	0.420	0.81 (0.69–0.94)	0.007
	*p* for trend		0.254		0.304		0.017

*Note:* SII: systemic immune‐inflammation index; Q2, Q3, Q4: interquartile; Q1 as reference. Model 1: adjusted for none. Model 2: adjusted for age, race, and gender. Model 3: adjusted for age, race, gender, smoke, HR, HbA1c, hypertension, stroke, diabetes, and congestive heart failure.

Abbreviations: CHD, coronary heart disease; CI, confidence intervals; LMR, lymphocyte‐to‐monocyte ratio; NLR, neutrophil‐to‐lymphocyte ratio; OR: odds ratio; PLR, platelet‐to‐lymphocyte ratio.

### 3.3. Correlation Analysis of Different Genders

To investigate the predictive value of gender‐specific inflammation indicators on the CHD population, we conducted a logistic regression analysis with gender stratification. The results (Table 4) indicated that LMR, NLR, PLR, and SII exhibited statistically significant associations with the occurrence of CHD in the male population under unadjusted Model 1 (all *p* < 0.05). In addition, higher NLR levels and lower LMR levels correlated with a higher incidence of CHD in both male and female groups (*p* < 0.001). When treated as categorical variables, the associations of LMR and NLR with CHD exhibited a trend consistent with the continuous variables in the two groups. In the male cohort, SII was found to promote CHD starting from Q4 (*p* = 0.002). Conversely, in the female cohort, the stratification of SII showed no statistical significance. In other models, such as the male Model 3 population, both categorical analyses of LMR, SII, and PLR consistently demonstrated statistically negative significance in relation to CHD (all *p* < 0.01), while NLR (*p* = 0.729) exhibited no statistical significance. In the female Model 3 population, we found that a significant negative correlation between LMR and CHD persists in the trend analysis (*p* for trend = 0.012). NLR was found to have a positive association with the risk of CHD in the female population (*p* < 0.05).

**Table 4:** Univariate and multivariate analyses between LMR/PLR/NLR/SII and CHD in different gender models.

		**Model 1**		**Model 2**		**Model 3**	
		**OR (95% CI)**	***p* value**	**OR (95% CI)**	***p* value**	**OR (95% CI)**	***p* value**
*Male*							
LMR	Continuous	0.73 (0.69–0.76)	< 0.001	0.93 (0.93–1.00)	0.082	0.99 (0.96–1.03)	0.723
	Q2 (2.8–< 3.6)	0.54 (0.46–0.62)	< 0.001	0.90 (0.76–1.06)	0.198	0.96 (0.81–1.15)	0.659
	Q3 (3.6–< 4.5)	0.34 (0.28–0.40)	< 0.001	0.71 (0.59–0.86)	< 0.001	0.79 (0.65–0.97)	0.025
	Q4 (≥ 4.5)	0.25 (0.21–0.30)	< 0.001	0.70 (0.58–0.85)	< 0.001	0.80 (0.65–0.99)	0.041
	*p* for trend		< 0.001		0.019		0.191

PLR	Continuous	1.00 (1.00–1.00)	0.005	1.00 (1.00–1.00)	0.005	1.00 (1.00–1.00)	0.028
	Q2 (91.8–< 115.6)	0.81 (0.69–0.96)	0.013	0.80 (0.67–0.96)	0.015	0.88 (0.73–1.07)	0.207
	Q3 (115.6–< 145.0)	0.70 (0.58–0.83)	< 0.001	0.65 (0.54–0.78)	< 0.001	0.73 (0.60–0.89)	0.002
	Q4 (≥ 145)	1.01 (0.87–1.19)	0.855	0.68 (0.57–0.81)	< 0.001	0.75 (0.63–0.91)	0.003
	*p* for trend		0.045		< 0.001		< 0.001

NLR	Continuous	1.25 (1.20–1.29)	< 0.001	1.06 (1.02–1.10)	0.002	1.01 (0.96–1.05)	0.729
	Q2 (1.5–< 2.00)	1.21 (0.98–1.49)	0.071	1.02 (0.82–1.27)	0.869	1.02 (0.81–1.29)	0.851
	Q3 (2.00–< 2.6)	1.71 (1.42–2.06)	< 0.001	1.12 (0.92–1.37)	0.269	1.04 (0.84–1.29)	0.690
	Q4 (≥ 2.6)	2.85 (2.39–3.39)	< 0.001	1.26 (1.04–1.52)	0.016	1.05 (0.85–1.29)	0.648
	*p* for trend		< 0.001		0.224		0.656

SII	Continuous	1.00 (1.00–1.00)	0.002	1.00 (1.00–1.00)	0.337	1.00 (1.00–1.00)	0.006
	Q2 (333.5–< 459.5)	0.84 (0.70–1.00)	0.054	0.78 (0.64–0.94)	0.008	0.73 (0.60–0.89)	0.002
	Q3 (459.5–< 636.2)	1.04 (0.88–1.23)	0.636	0.84 (0.70–1.01)	0.065	0.83 (0.68–1.01)	0.056
	Q4 (≥ 636.2)	1.28 (1.09–1.50)	0.002	0.79 (0.66–0.94)	0.007	0.67 (0.55–0.81)	< 0.001
	*p* for trend		0.354		0.003		< 0.001

*Female*							
LMR	Continuous	0.74 (0.70–0.79)	< 0.001	0.91 (0.85–0.96)	0.001	0.94 (0.89–1.00)	0.057
	Q2 (3.2–< 4.0)	0.55 (0.43–0.69)	< 0.001	0.78 (0.61–0.99)	0.041	0.94 (0.72–1.21)	0.621
	Q3 (4.0–< 5.2)	0.37 (0.29–0.46)	< 0.001	0.64 (0.50–0.81)	< 0.001	0.78 (0.60–1.01)	0.058
	Q4 (≥ 5.2)	0.34 (0.27–0.43)	< 0.001	0.72 (0.56–0.94)	0.014	0.85 (0.64–1.12)	0.242
	*p* for trend		< 0.001		< 0.001		0.012

PLR	Continuous	1.00 (1.00–1.00)	0.240	0.99 (0.99–1.00)	0.234	1.00 (1.00–1.00)	0.390
	Q2 (98.9–< 123.5)	0.68 (0.53–0.86)	0.002	0.70 (0.55–0.90)	0.006	0.75 (0.58–0.98)	0.032
	Q3 (123.5–< 155.5)	0.72 (0.57–0.92)	0.008	0.72 (0.56–0.92)	0.009	0.79 (0.61–1.02)	0.071
	Q4 (≥ 155.5)	0.90 (0.72–1.13)	0.357	0.73 (0.58–0.92)	0.008	0.80 (0.63–1.03)	0.079
	*p* for trend		0.401		0.037		0.431
NLR	Continuous	1.21 (1.16–1.27)	< 0.001	1.13 (1.07–1.18)	< 0.001	1.06 (1.00–1.12)	0.049
	Q2 (1.5–< 2.0)	1.07 (0.82–1.39)	0.639	1.00 (0.76–1.31)	0.974	0.94 (0.71–1.25)	0.666
	Q3 (2.0–< 2.6)	1.31 (1.02–1.69)	0.037	1.18 (0.91–1.54)	0.213	1.04 (0.79–1.37)	0.797
	Q4 (≥ 2.6)	1.80 (1.42–2.28)	< 0.001	1.35 (1.05–1.73)	0.020	1.02 (0.78–1.33)	0.868
	*p* for trend		< 0.001		0.006		0.237

SII	Continuous	1.00 (1.00–1.00)	< 0.001	1.00 (1.00–1.00)	0.003	1.00 (1.00–1.00)	0.293
	Q2 (365.7–< 505.1)	0.89 (0.69–1.15)	0.375	0.90 (0.70–1.17)	0.447	0.96 (0.73–1.26)	0.771
	Q3 (505.1–< 710.0)	1.12 (0.88–1.41)	0.369	1.15 (0.90–1.47)	0.256	1.07 (0.83–1.39)	0.592
	Q4 (≥ 710.0)	1.18 (0.93–1.49)	0.175	1.16 (0.91–1.48)	0.235	0.99 (0.76–1.28)	0.925
	*p* for trend		0.090		0.053		0.289

*Note:* SII: systemic immune‐inflammation index; Q2, Q3, and Q4: interquartile; Q1 as reference. Model 1: adjusted for none. Model 2: adjusted for age and race. Model 3: adjusted for age, race, smoke, HR, HbA1c, hypertension, stroke, diabetes, and congestive heart failure.

Abbreviations: CHD, coronary heart disease; CI, confidence intervals; LMR, lymphocyte‐to‐monocyte ratio; NLR, neutrophil‐to‐lymphocyte ratio; OR, odds ratio; PLR, platelet‐to‐lymphocyte ratio.

### 3.4. RCS Regression and ROC Curve Analysis of Inflammation Markers and CHD in Different Genders

Next, we used RCS regression to visualize the correlation of predicted inflammation markers and CHD risk in the male and female population. The risk of CHD in the male group was relatively flat until around 3.57 of predicted LMR and then started to increase afterward (*p* for nonlinearity = 0.042, Figure 2(a)). We did not observe a nonlinear correlation between NLR and CHD (*p* for nonlinearity = 0.233) in the male group. The strong U‐shaped relation between predicted PLR and OR value of CHD risk in the male group, the plot, showed a substantial increase of the CHD risk within the lower range of predicted PLR, which reached the critical juncture around 111.85 and then decreased gradually (*p* for nonlinearity < 0.001). Before reaching the critical point of 448.12, SII exhibited a positive influence on the promotion of CHD occurrence, albeit with a gradually diminishing effect. Beyond this threshold, the impact shifts toward slightly preventing the risk of CHD (*p* for nonlinearity < 0.001).

Figure 2The analysis of restricted cubic spline regression (a) and (b) represented different sex groups adjusted for age, race, smoke, HR, HbA1c, hypertension, stroke, diabetes, and congestive heart failure; SII: systemic immune‐inflammation index; NLR: neutrophil‐to‐lymphocyte ratio; PLR: platelet‐to‐lymphocyte ratio; LMR: lymphocyte‐to monocyte ratio.

**Figure:**
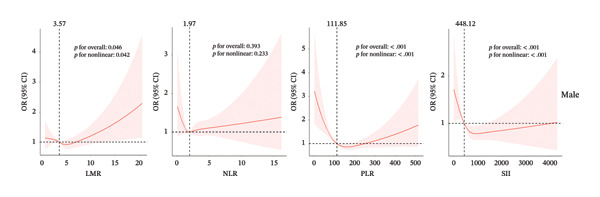
(a)

**Figure:**
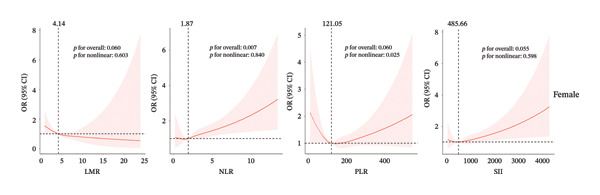
(b)

For the female cohort (Figure 2(b)), the nonlinear association between LMR, NLR, and SII with CHD showed no statistical significance (all *p* for nonlinearity > 0.05). Only PLR exhibited a consistent U‐shaped trend similar to that observed in the male cohort (*p* for nonlinearity = 0.025). The ROC curve (Figure 3) was used to determine the predictive value for SII, LMR, NLR, and PLR with CHD. Among patients with male (AUC = 0.658) or female (AUC = 0.627), LMR demonstrated superior predictive value for CHD compared with SII, NLR, and PLR.

Figure 3The analysis of the receiver operating characteristic curve. Comparison of the predictive value of SII, LMR, NLR, and PLR for the male group (a) and female group (b). The area under the curve (AUC) for assess the model’s predictive power. SII: systemic immune‐inflammation index; NLR: neutrophil‐to‐lymphocyte ratio; PLR: platelet‐to‐lymphocyte ratio; LMR: lymphocyte‐to‐monocyte ratio.

**Figure:**
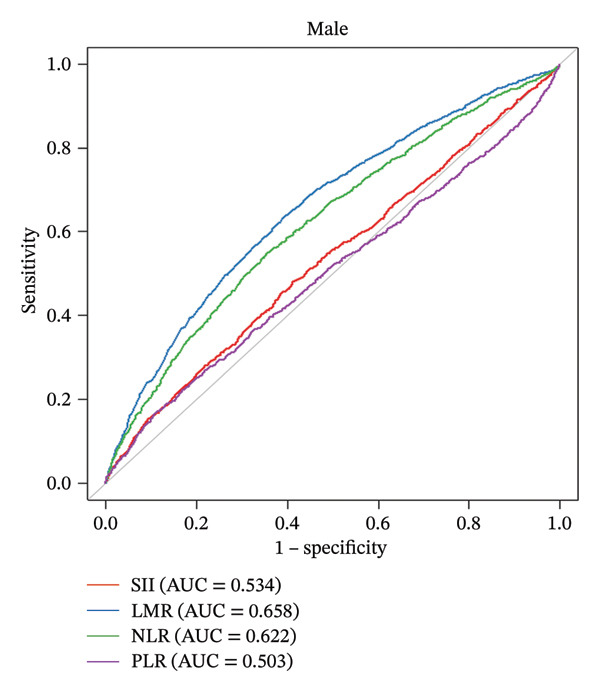
(a)

**Figure:**
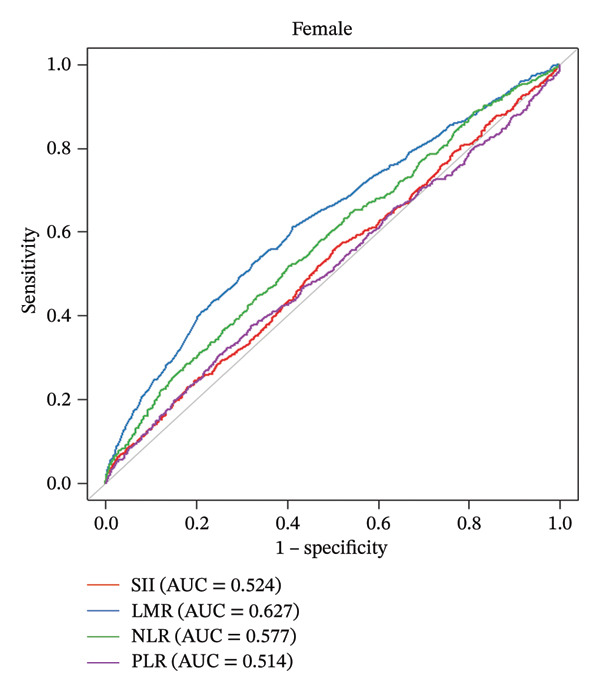
(b)

## 4. Discussion

By analyzing 16‐year population survey data from the NHANES database, we observed significant differences in multiple inflammatory biomarkers, including LMR, PLR, NLR, and SII between individuals with or without CHD.

After normalizing for various confounding factors, we revealed that SII, LMR, and PLR exhibited a negative correlation with the incidence of CHD in the entire population, while NLR showed a positive correlation with CHD. We further stratified the study by sex and observed that SII, LMR, and PLR remained as independent risk factors for CHD in the male population, whereas LMR and NLR emerged as an independent risk factor for CHD in the female population. In addition, we observed a nonlinear correlation between SII, LMR, and PLR with the risk of CHD in the male population; however, only PLR exhibited a nonlinear relationship with the risk of CHD in the female population.

In recent years, numerous studies have been conducted to investigate the correlation between inflammatory factors and cardiovascular disease. For example, Núñez et al. discovered that the NLR served as a superior and more effective biomarker for predicting subsequent mortality in ST elevation myocardial infarction (STEMI) patients compared to WBC [18]. Similarly, in a study of patients with non‐ST elevation myocardial infarction (NSTEMI), NLR > 3.88 was found to be independently and positively associated with the frequency of myocardial infarction recurrence [19]. In our study, NLR is significantly positively associated with CHD in both the entire population and the female in sex‐stratified subgroups. We believe that the prognostic value of NLR is more pronounced in the female cohort. In a prospective study examining the relationship between NLR and all‐cause mortality and cardiovascular mortality, it was also observed that NLR exhibited an association with CHD. Furthermore, when NLR exceeded 1.77, there was a higher prevalence of mortality among male in the general population [20]. Higher NLR levels might serve as a reliable indicator for an increased incidence of CHD diagnosis in the male demographic. The relationship between LMR and cardiovascular disease is intricate. Gary et al. observed a significant association between low LMR in patients with peripheral arterial occlusive disease and an increased risk of acute limb ischemia [21]. In STEMI patients, positive correlation was observed between LMR and PCI recovery, with lower LMR levels associated with poorer coronary flow recovery [22]. However, studies have also indicated that LMR serves as an independent risk factor for CHD and exhibits a positive correlation with the extent of coronary artery stenosis [9]. Our multimodel regression study found that LMR and CHD were inversely correlated in the general population (Model 1 and Model 2). In Model 3, the LMR stratification analysis for Q3 and Q4 continues to exhibit a significant negative correlation with CHD. Thus, we propose a stronger negative correlation between LMR and CHD in males.

The process of blood cell–induced inflammation leading to atherosclerotic plaques is often complex and diverse. Leukocytes that migrate and adhere to the inner surface of the artery wall play a key role in the early stages of vascular plaque formation [23]. Clinical studies also demonstrated that white blood cell counts were an independent predictor of CHD risk, with high neutrophil counts or low lymphocyte counts offering greater predictive power [24]. Monocytes and macrophages infiltrate the arterial walls in response to endothelial injury, where they differentiate and contribute to plaque formation by secreting pro‐inflammatory cytokines, such as TNF‐α and IL‐6 [25]. Platelets support the concentration of white blood cells at the site of inflammation. At the same time, they perform a hemostatic function by ensuring the integrity of inflamed blood vessels to prevent bleeding from permeating the site of white blood cells [26]. In CHD patients in this research, inflammatory markers, such as white blood cells, neutrophils, and monocytes, were significantly higher, while platelets were significantly lower compared to non‐CHD patients (Table 1). Further study is warranted to explore the role of these inflammatory cytokines in different sex groups.

Recently, the SII inflammatory indicator has been found to be important in predicting the risk of various cardiovascular diseases. SII integrates the interaction of various inflammatory cells, elucidates their role in disease pathophysiology, and serves as a powerful indicator of systemic immune inflammatory state and prognosis [27]. Studies have shown that SII can effectively predict the long‐term risk of death from heart failure with reduced ejection fraction (HFrEF) in patients with intracardiac defibrillators [28]. SII also plays a significant role as a diagnostic factor in heart failure patients with renal insufficiency [29]. Zhang and Chen discovered that patients with higher SII levels faced a greater risk of peripheral vascular disease [30]. A NHANES study suggested that higher SII values may be linked to a greater incidence of CHD. Furthermore, smooth curve fitting analysis showed an inverted U‐shaped relationship between SII and CHD risk in the female group, but no significant nonlinear relationship was found in the male group [8]. Our study contradicts the results of previous research. In the male population, we observed that SII facilitated the risk of CHD at the early stage but began to exhibit inhibitory effects at the advanced stage (Q4). In the female population, there was no statistically significant nonlinear association between SII and CHD.

We realized that there are some limitations of this research. Firstly, all laboratory data are limited to a single cross‐sectional observation in one country, lacking longitudinal tracking data and other region data. Secondly, all CHD data were obtained through questionnaires, and their authenticity requires further investigation, potentially impacting subsequent results. Lastly, there is a need for further expansion of the sample size.

## 5. Conclusion

Our study reveals that LMR, PLR, and SII are negatively significant markers of CHD in males, while LMR and NLR are superior predictors of CHD in females. This finding holds significant implications for the clinical prevention and treatment of CHD, warranting further investigation into the relationship between inflammatory markers and CHD in future studies.

## Author Contributions

Anmin Ren, Qianjun Liu, and Qian Gan contributed to the hypothesis development and the manuscript preparation; Liming Lu contributed to the language refinement; Xin Kai Qu contributed to the manuscript revision.

## Funding

No specific funding was received for this work.

## Disclosure

All authors read and approved the final manuscript.

## Ethics Statement

The NHANES research protocols were approved by the NCHS Research Ethics Review Board, and all participants provided written informed consent.

## Conflicts of Interest

The authors declare no conflicts of interest.

## Data Availability

The data that support the findings of this study are available in NHANES at https://www.cdc.gov/nchs/nhanes/index.htm.
